# Synergistic effect of microneedle-delivered extracellular matrix compound and radiofrequency on rejuvenation of periorbital wrinkles

**DOI:** 10.3389/fmed.2022.900784

**Published:** 2022-07-22

**Authors:** Haiyan Cheng, Ruina Zhang, Fenglin Zhuo

**Affiliations:** Department of Dermatology, Beijing Friendship Hospital, Capital Medical University, Beijing, China

**Keywords:** microneedle, extracellular matrix, radiofrequency, periorbital wrinkles, skin biophysical parameters

## Abstract

**Background:**

A combination of minimally invasive modalities can induce collagen regeneration more quickly and promote the penetration of topical agents, thus promoting skin rejuvenation. In this study, we aimed to investigate the synergistic efficacy of extracellular matrix compound (ECM-C) *via* microneedle (MN) and radiofrequency (RF) on periorbital wrinkles.

**Method:**

A total of 25 participants with periorbital wrinkles were selected for this study. The left and right side of the periorbital area was randomly given ECM-C *via* MN or ECM-C *via* MN combined with RF. MN combined with ECM-C treatment was given 5 times at 2 weeks intervals, whereas RF treatment was given 3 times at 4-week intervals. The following items were assessed: wrinkles by VISIA^®^ system; biophysical parameters such as skin hydration, transepidermal water loss (TEWL), erythema index, and melanin index by CK multiple probe adapter; and skin elasticity and skin thickness by DermaLab Combo^®^ photographs were taken at the baseline and 2 weeks after the last treatment. Subjective assessments, such as Crow's Feet Grading Scale (CFGS) and Global Aesthetic International Scale (GAIS), were also recorded.

**Result:**

A total of 25 participants with an average age of 43 years participated in this trial. Periorbital wrinkles on both sides decreased after the treatment, and the side treated with ECM *via* MN and RF showed better improvement than the other side with ECM-C *via* MN alone. Skin hydration increased after the treatment on both sides. TEWL, skin erythema, and skin melanin indexes were not changed. Skin elasticity and skin thickness increased more on the side of ECM-C *via* MN and RF than on the other side of ECM-C *via* MN alone. The evaluation scores for CFGS improved on either side; however, no difference was found for CFGS and GAIS between intergroup comparisons after the treatment.

**Conclusion:**

The objective assessment of wrinkles, elasticity, and thickness of periorbital skin improved more on the side with ECM-C treatment *via* MN combined with RF than on the other side of ECM-C treatment *via* MN only. However, no statistically significant difference was found between the subjective CFGS and GAIS evaluation of the two sides.

## Introduction

Periorbital aging, including eyelid drooping and the appearance of fine lines and wrinkles, is usually the first to occur because the area around the eyes has the thinnest layer and is exposed the most (1, 2). Invasive and non-invasive techniques are used to rejuvenate periorbital wrinkles, laxity, and abnormal pigmentation (3, 4). Non-invasive and minimally invasive methods are widely accepted because of lesser pain, post-treatment complications, and downtime and give the desired effect. Among them, microneedle (MN) treatment is performed using fixed sterile needles that penetrate the skin to the dermis, which creates many minimum prick wounds. This initiates inflammation and wound healing process and causes the deposition of collagen and elastin. The channel penetrated by MN facilitates the delivery of topical cosmetic products to increase the aesthetic outcome (5–7).

Extracellular matrix (ECM), including collagen, elastin, hyaluronic acid, laminin, fibrin, and integrins, helps skin tissue to maintain homeostasis and youthful appearance (8). Nowadays, many cosmetic products are focusing on the ECM components to achieve facial rejuvenation effect. These products are beneficial for repairing the broken collagen, restoring new collagen, and hydrating the tissue to help maintain the skin firm and moist (9–11). In this study, ECM-C, a product containing multiple ECM components, such as types I/III/IV/VII collagen, laminin, nidogen, and hyaluronic acid, helped to restore the firm skin barrier to sustain a youthful appearance.

Radiofrequency (RF) was effective for periorbital wrinkles in the long term, which induced collagen remodeling by heating (12). Nevertheless, only a few research studies report the effect of the combination of MN, ECM-C, and RF treatment for periorbital rejuvenation.

In this study, we investigated the synergistic effect of ECM-C *via* MN and RF on periorbital wrinkles. The changes in biophysical parameters after this treatment, along with the satisfaction and adverse effect, were recorded.

## Materials and methods

### Study design

This study is a single-center, randomized, face-split research conducted from September to December 2018. A total of 25 participants were recruited for this study on periorbital rejuvenation. The patients who drew an odd number were assigned to be given the roller MN (0.75-mm needle length, Xiunuo Photoelectricity Technology Limited Company, China) combined with ECM-C (MEL JIN, KSG Biotechnology Company, Beijing) treatment every 2 weeks for five times on the left periorbital area, and the other side wound was provided an additional RF treatment (Thermo Lift Radio Frequency, Alma, USA) for three times at 4-week interval. The patients who drew an even number were assigned to be given ECM-C *via* MN for the right periorbital area, and the other side wound was provided an additional RF treatment. Subjective and objective assessments were recorded before and 2 weeks after the last treatment. This research was conducted in accordance with the ethical guidelines of the Declaration of Helsinki and was permitted by the institutional review board of the Beijing Friendship Hospital Ethics Committee. All participants provided their signed informed consent and were allowed publication of their photographs before the initiation of this study (clinical trial number: ChiCTR1900021425).

### Population selection

Participants were included if i) they were of age 35–65 years with intact facial skin; ii) their periorbital skin manifested the symptoms of aging, laxity, fine wrinkle, or hyperpigmentation; iii) they had no filler treatment or the application of hyaluronic acid-associated medical cosmetic products or laser/RF/focused ultrasound treatment on the face in the latest 6 months; and iv) they had not used oral steroids, collagen, hyaluronic acid, and relative cosmetical products or skin-boosting supplements in the last 3 months. Patients suffering from endangering diseases or who were unable to cooperate with the entire treatment scheme and follow-up were excluded.

### Objective assessments

Before the first treatment and at 2 weeks after the last treatment, all participants were asked to clean their face with clean water and dry it with soft cloth tissue, followed by resting for 30 min in a quiet room. Next, photographs were taken by the VISIA Skin Analysis System (Canfield Company, USA), and the presence of periorbital wrinkles was analyzed. Then, skin hydration, transepidermal water loss (TEWL), skin melanin, and erythema indexes 2 cm down the lateral canthus for each eye were tested with the CK multiple-probe adapter (Courage+Khazaka Electronic GmbH, Germany) in a room with a constant room temperature at 24°C and a room humidity of 50%. The measurements were performed three times to obtain a mean value. Finally, skin elasticity and thickness were determined at the same site by the DermaLab Combo^®^ (Cortex Technology, Hadsund, Denmark), and the ultrasound images were recorded. ViscoElasticity (VE) represents skin elasticity, which was calculated from both the elevation phase and retraction phase. Skin thickness (recorded in um) indicated the thickness of the dermis, as determined by the intensity of the reflected ultrasound.

### Treatment procedure

For the side treated with MN plus ECM-C, 5% compound lidocaine cream (Tongfang Pharmaceutical Group Company) was applied topically for 40 min, after which the performer sterilized the area of the eyes with 75% alcohol. Next, 0.5 ml of ECM-C was rubbed around the periocular skin, and another 0.5 ml of ECM-C was allowed to penetrate the periocular skin following MN treatment. For the side treated with RF and MN plus ECM-C, the periocular skin first received RF treatment at the power of 80 W, and the whole energy for the periocular area was 15 J/cm^2^. After 10 min of cooling, the abovementioned process of ECM-C *via* MN was performed.

### Subjective assessment

Two dermatologists who were blinded to this trial assessed the scores according to the four-point Crow's Feet Grading Scale (CFGS). The scores were recorded on a range of 0–4, with 0 = no wrinkles, 1 = very fine wrinkles, 2 = fine wrinkles, 3 = moderate wrinkles, and 4 = severe wrinkles.

As for the participants, the five-point Global Aesthetic Improvement Scale (GAIS) questionnaire was used to assess their satisfaction with the outcomes, which was scored on a range from −1 to 3, with −1 = worse than the baseline, 0 = no change, 1 = improved, 2 = much improved, and 3 = very much improved.

### Adverse-effect assessment

The scores of the visual analog scale (VAS) for pain were recorded during the period of MN treatment for the first time. Adverse effects occurring during and after the treatment were noted, which included unbearable pain, burn, bruise, bleeding, lump, skin irritation, redness, and itch.

### Statistical analysis

All data were analyzed by the SPSS 17.0 (Chicago, USA), a paired-sample *t*-test was used to compare the efficacy at the baseline and follow-up visit on the same side of the face, and an independent samples *t*-test was used to compare two sides. *p* < 0.05 was considered to indicate statistical significance.

## Results

### Population

A total of 25 Asian female volunteers participated in this study, whose ages ranged from 35 to 65 years (median age: 43 years). A total of 18 volunteers had Fitzpatrick skin type IV and 7 volunteers had Fitzpatrick skin type III.

### Objective tests

#### CK tests

**Figure 1:**
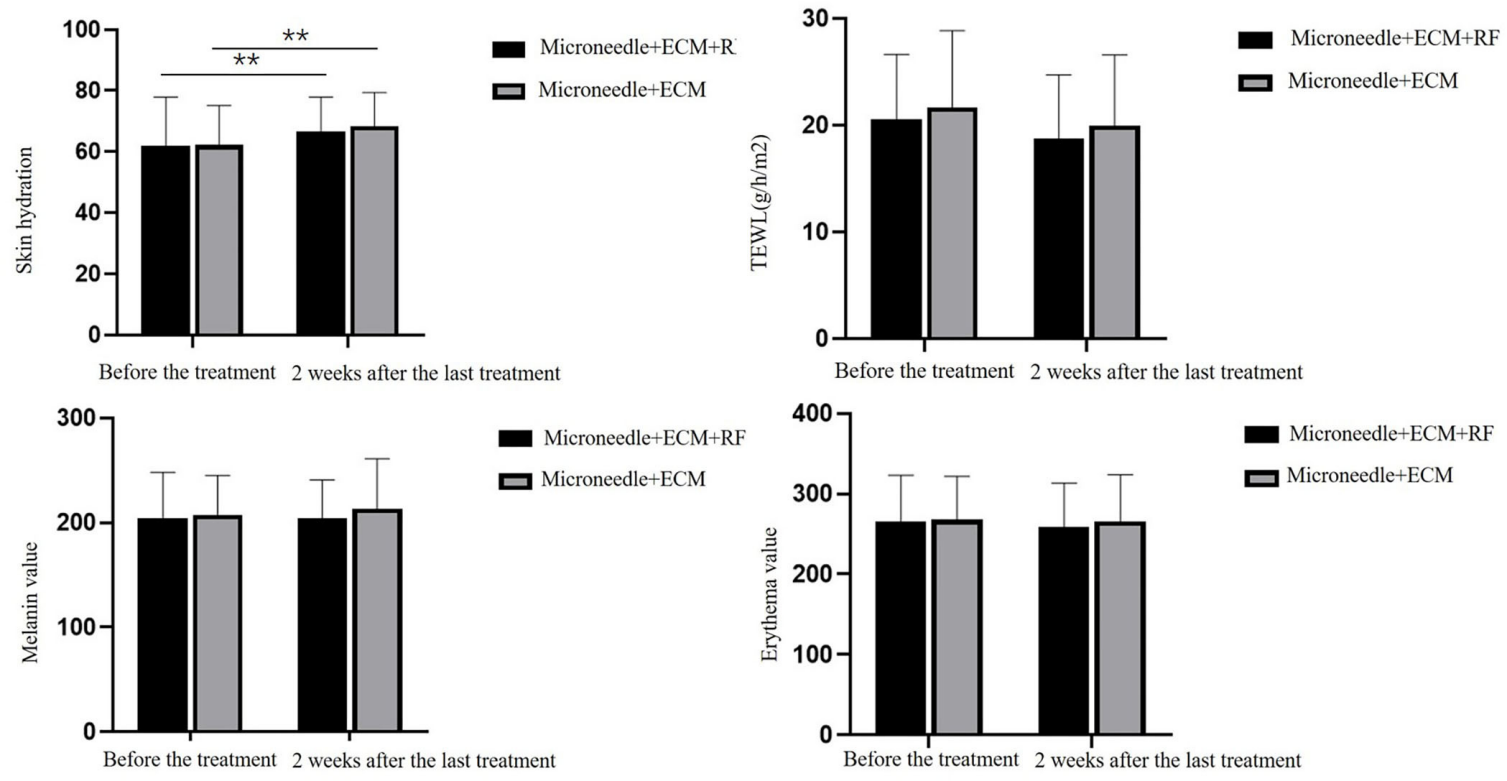
Comparison graph of biophysical parameters measured by CK. TEWL, transepidermal water loss. ** *p* < 0.01.

The CK values, including skin hydration, TEWL, melanin index, and erythema index, before and 2 weeks after the last treatment are shown in Table 1 and Figure 1. Skin hydration value increased either on the side of ECM-C *via* MN only (*p* < 0.01) or on the other side of ECM-C *via* MN along with RF (*p* = 0.01) 2 weeks after the last treatment. No significant difference was found between the two sides, 2 weeks after the last treatment (*p* = 0.18). TEWL, melanin index, and erythema index were not significantly different on either side (*p* = 0.14 and *p* = 0.16) or between the two sides (*p* = 0.08), 2 weeks after the last treatment.

**Table 1:** Comparison of the results of biophysical parameters measured by CK.

	**Before the treatment**	**2 weeks after the last treatment**	***t*** **-value**	***p-*** **value**
**TSkin hydration** Microneedle+ECM+RF Microneedle+ECM *t*-value *p*-value	61.99 ± 15.58 62.31 ± 12.83 0.23 0.82	66.54 ± 11.36 68.41 ± 11.00 1.38 0.18	−2.72 −4.56	**0.01** **<0.01**
**TEWL (g/h/m^**2**^)** Microneedle+ECM+RF Microneedle+ECM *t*-value *p*-value	20.53 ± 6.11 21.66 ± 7.19 1.84 0.08	18.71 ± 6.01 19.97 ± 6.62 1.85 0.08	1.54 1.45	0.14 0.16
**Melanin value** Microneedle+ECM+RF Microneedle+ECM *t*-value *p*-value	204.56 ± 43.46 207.52 ± 37.52 0.56 0.58	204.02 ± 36.81 213.40 ± 47.62 1.57 0.13	0.08 −0.99	0.94 0.33
**Erythema value** Microneedle+ECM+RF Microneedle+ECM *t*-value *p*-value	265.78 ± 57.52 267.76 ± 54.12 0.27 0.79	259.00 ± 54.66 265.82 ± 58.07 0.86 0.4	1.09 0.23	0.29 0.82

Statistics are presented as mean ± SD. TEWL, transepidermal water loss. Statistical different *p* values were shown in bold.

#### VISIA skin analysis system

**Figure 2:**
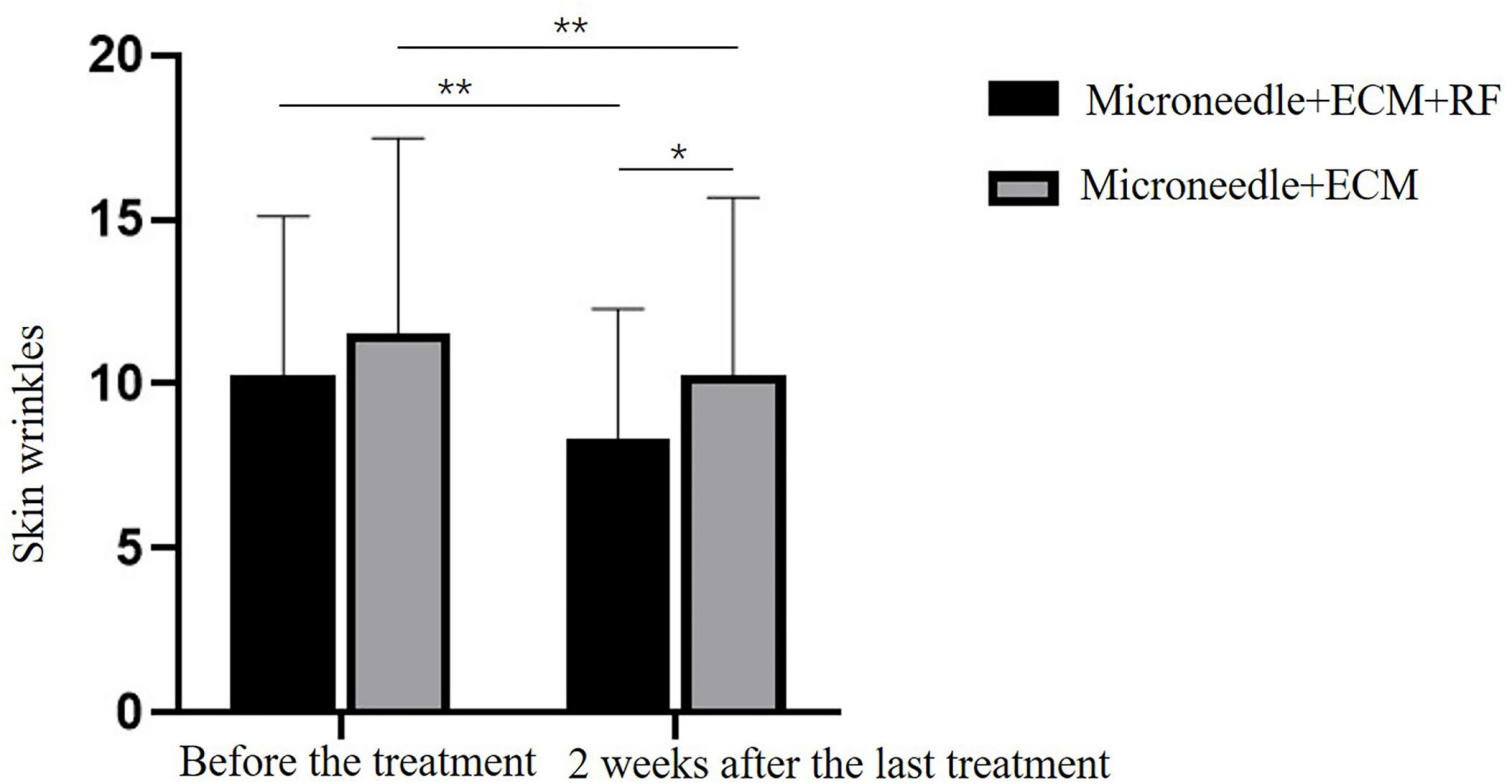
Comparison graphs of periorbital wrinkles measured by VISIA. *p < 0.05, **p < 0.01.

**Figure 3:**
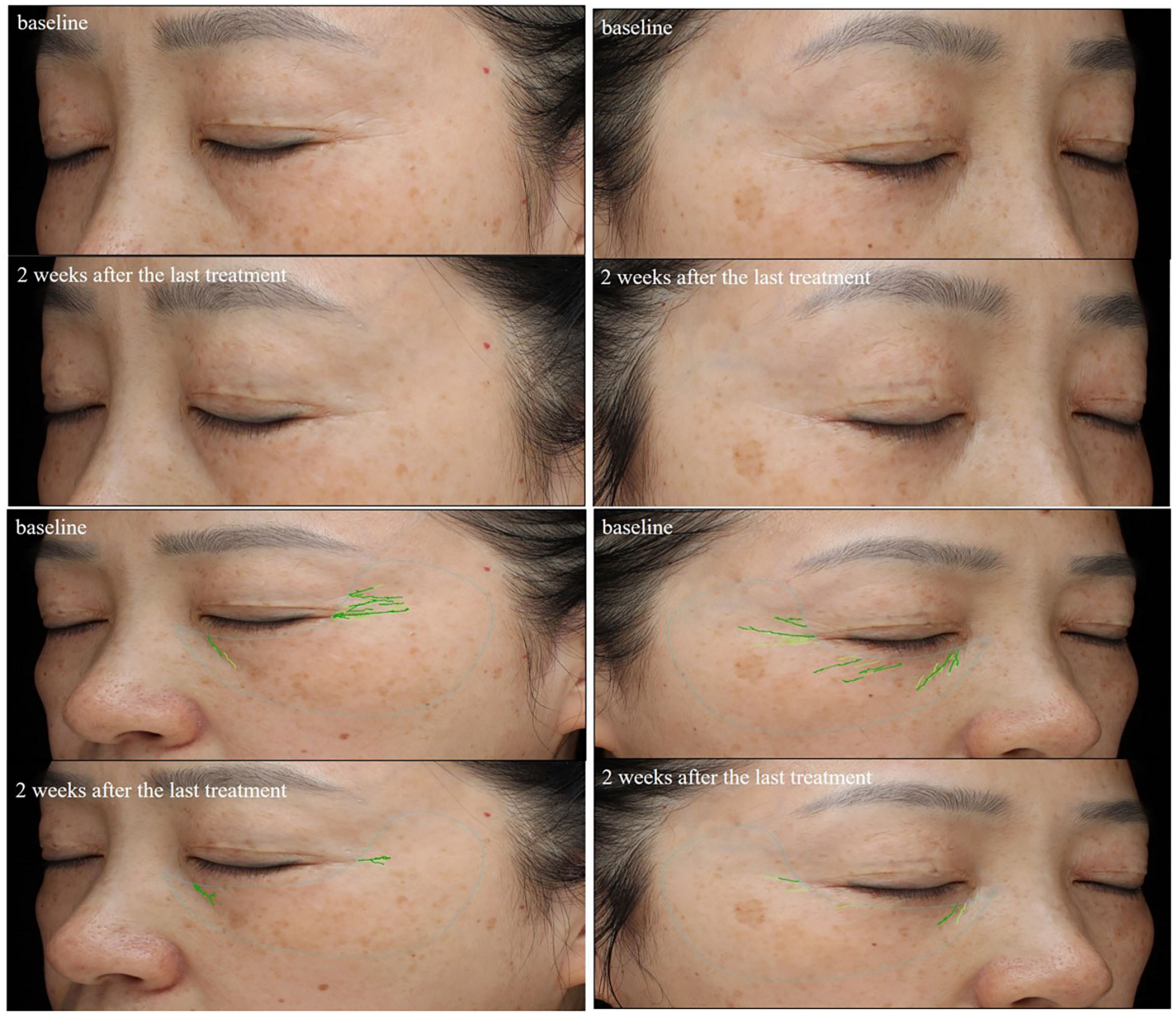
Comparison of the periorbital wrinkles in a 49-year-old woman based on the VISIA images. Her left periorbital area was treated with ECM-C *via* MN; the wrinkles score assessed by the VISIA images system decreased from 5.55 to 1.84; her right periorbital area was treated with ECM-C *via* MN and radiofrequency; the wrinkles score assessed by the VISIA images system decreased from 6.26 to 1.13.

**Figure 4:**
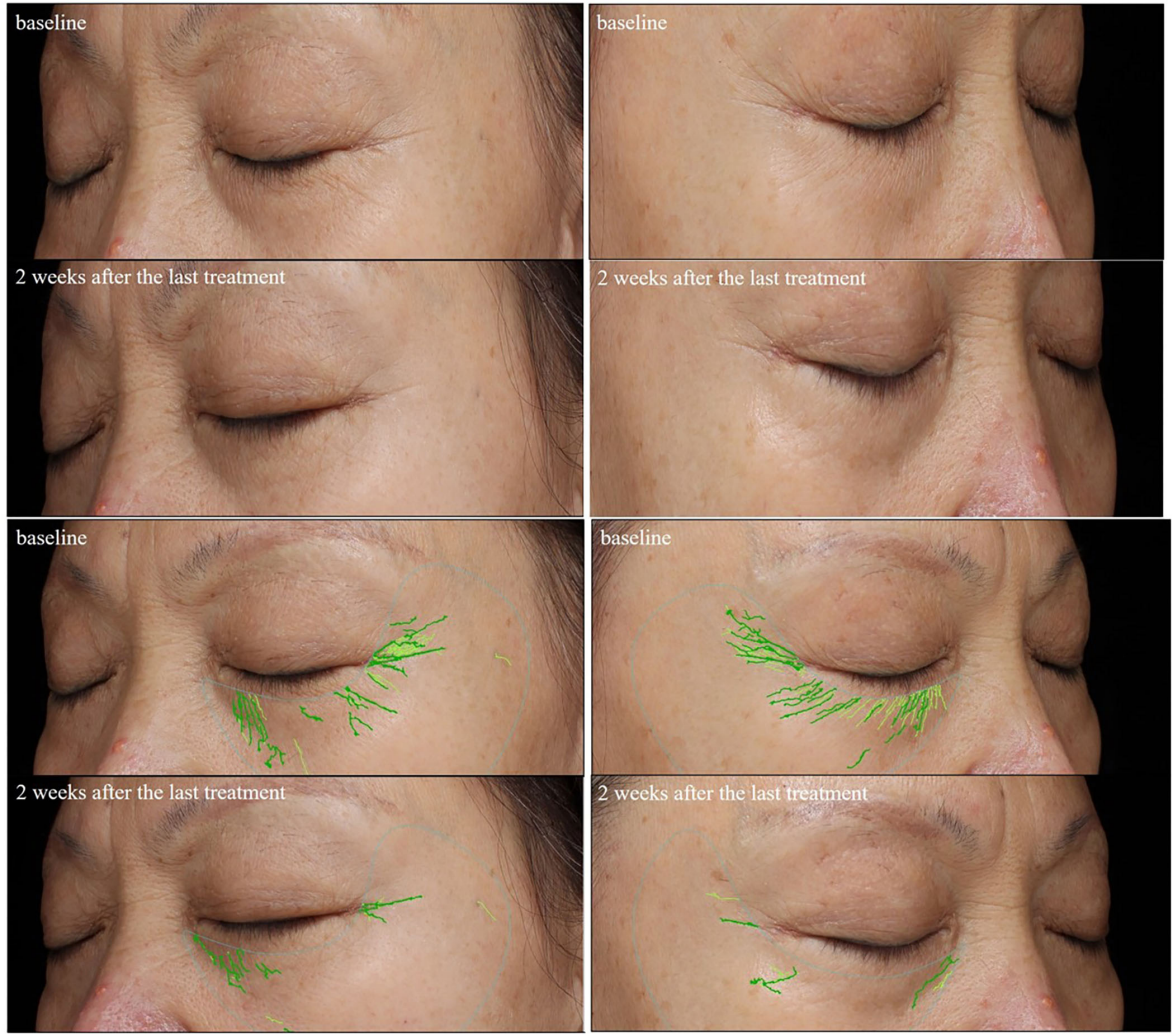
Comparison of the periorbital wrinkles in a 59-year-old woman with the VISIA images system. Her left periorbital area was treated with ECM-C *via* MN; the wrinkles score assessed by the VISIA images system decreased from 13.54 to 5.78; her right periorbital area was treated with ECM-C *via* MN and radiofrequency; the wrinkles score assessed by the VISIA images system decreased from 17.37 to 3.80.

The results of periocular wrinkles measured by VISIA are presented in Table 2 and Figure 2. Compared with baseline, ECM-C *via* MN along with RF and the other side of ECM-C *via* MN only showed decreased scores of periocular wrinkle 2 weeks after the last treatment (*p* < 0.01 and *p* < 0.01). The scores of periocular wrinkles 2 weeks after the last treatment decreased significantly more at the side of ECM-C *via* MN along with RF than on the side of ECM-C *via* MN only (*p* = 0.03). The images are shown in Figures 3, 4.

**Table 2:** Comparison of the results of periorbital wrinkles as measured by the VISIA system.

	**Before the treatment**	**2 weeks after the last treatment**	***t*** **-value**	***p-*** **value**
**Skin wrinkles** Microneedle+ECM+RF Microneedle+ECM *t*-value *p*-value	10.29 ± 4.82 11.57 ± 5.90 1.37 0.19	8.33 ± 3.96 10.29 ± 5.38 2.28 **0.03**	6.34 4.15	**<0.01** **<0.01**

Statistics are presented as the mean ± SD. Skin wrinkles were measured around the periorbital area symmetrically. Statistical different *p* values were shown in bold.

#### UBM assessment

**Figure 5:**
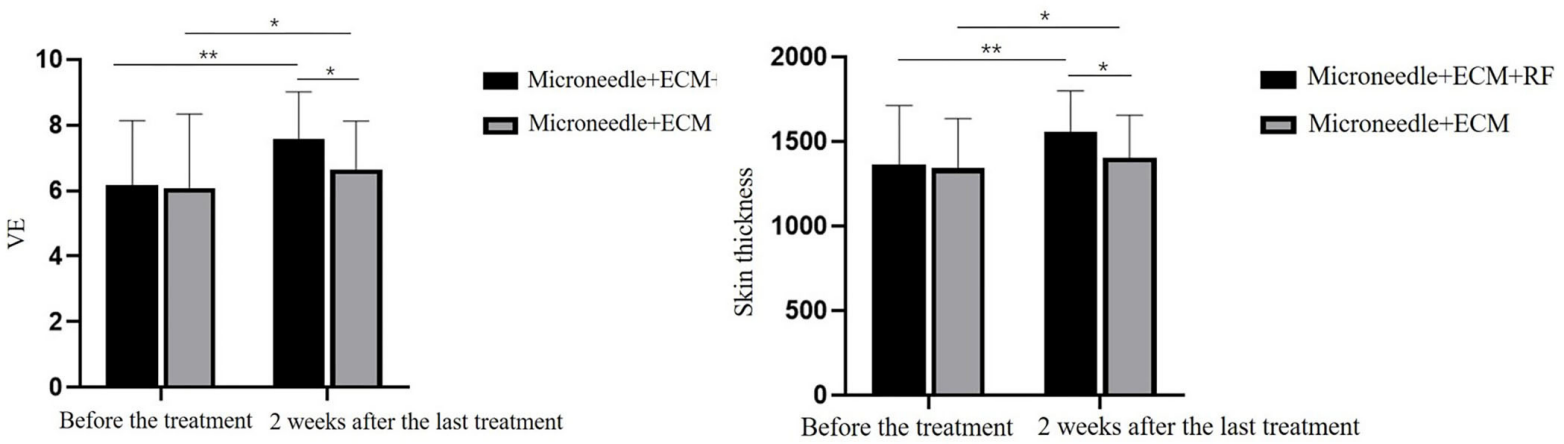
Comparison graphs of skin elasticity and thickness measured by UBM. * *p* < 0.05, ** *p* < 0.01.

Skin elasticity and thickness were measured by UBM, and the comparison results are shown in Table 3 and Figure 5. Compared with baseline, both the sides of the two intervenes showed statistically significant changes in the scores for skin elasticity and skin thickness 2 weeks after the last treatment (*p* < 0.01 and *p* < 0.01 for the side treated with ECM-C *via* MN and RF, *p* = 0.04 and *p* = 0.04 for the side treated with ECM-C *via* MN). The side treated with ECM-C *via* MN and RF showed better improvement in skin elasticity and thickness than the other side (*p* = 0.03 and *p* = 0.03).

**Table 3:** Comparison of the results of skin elasticity and skin thickness by UBM.

	**Before the treatment**	**2 weeks after the last treatment**	***t*** **-value**	***p-*** **value**
**VE** ECM-C via MN + RF ECM-C via MN *t*-value *p* value	6.09 ± 1.95 6.09 ± 2.25 0.00 1.00	7.59 ± 1.42 6.65 ± 1.47 −2.31 **0.03**	−7.20 −2.15	**<0.01** **0.04**
**Skin thickness (um)** ECM-C via MN+RF ECM-C via MN *t*-value *p*-value	1365.16 ± 346.59 1342.48 ± 292.65 −0.25 0.80	1557.54 ± 241.99 1403.39 ± 252.36 −2.21 **0.03**	−6.20 −2.23	**<0.01** **0.04**

Statistics are presented as the mean ±SD. VE represents skin elasticity; the greater the number of VE, the better the skin elasticity. Statistical different *p* values were shown in bold.

**Figure 6:**
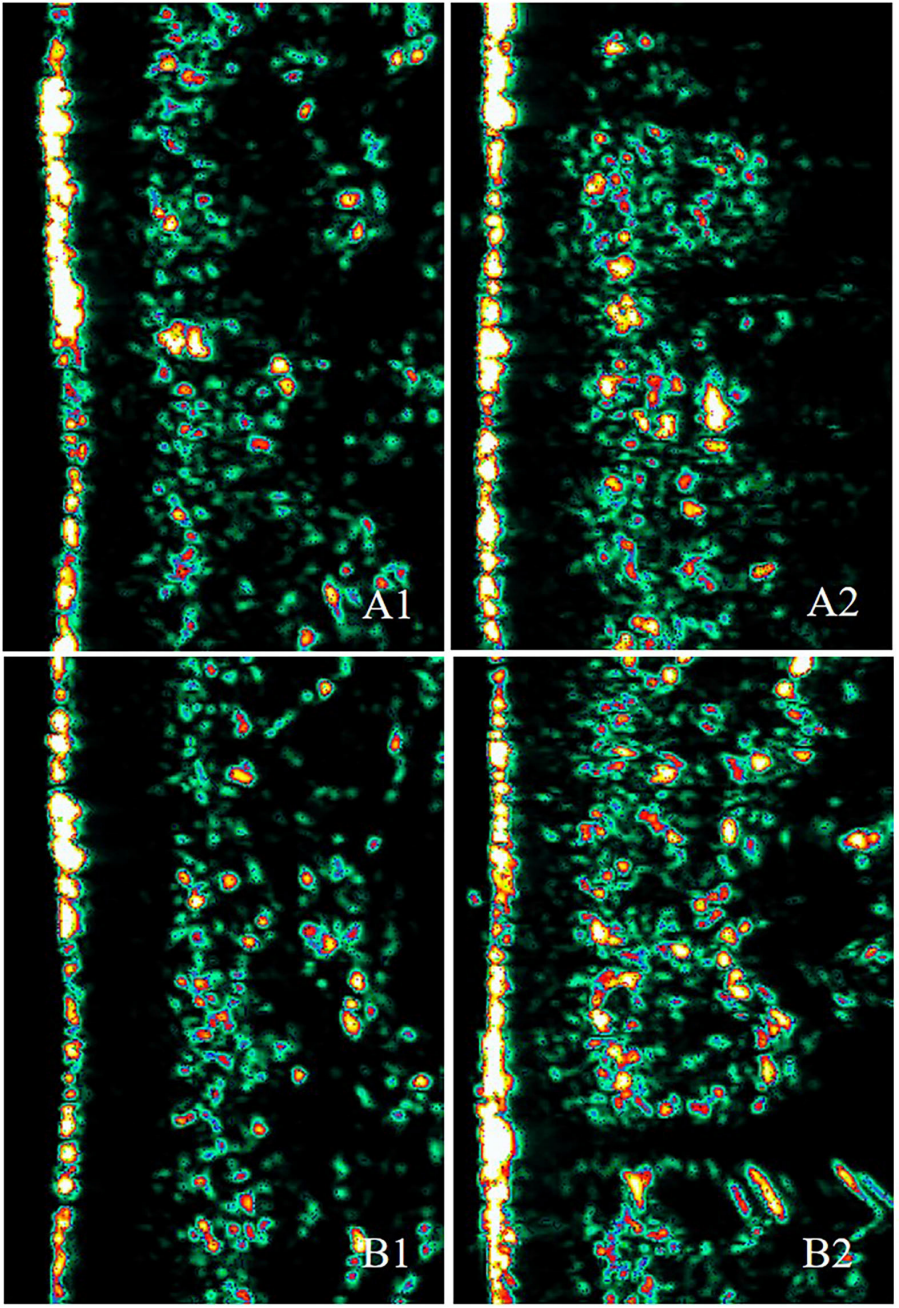
Ultrasound images of a 40-year-old woman at the baseline and at 2 weeks after the last treatment. A1 and B1 show images taken at the baseline (A1: skin elasticity = 5.3, skin thickness = 1,290.3 μm; B1: skin elasticity = 5.3, skin thickness = 1,158.7 μm). A2 shows the image taken 2 weeks after the last ECM-C *via* MN treatment (skin elasticity = 5.6, skin thickness = 1,422 μm). B2 represents the image taken 2 weeks after the last ECM-C *via* MN and RF treatment (skin elasticity = 6.1, skin thickness = 1,457 μm). Echogenicity color scale: white>yellow>red>green>black.

The ultrasound images of the skin dermis are also shown in Figure 6. Echogenicity increased for two different treatments, 2 weeks after the last treatment.

### Subjective assessment

#### CFGS assessment

**Figure 7:**
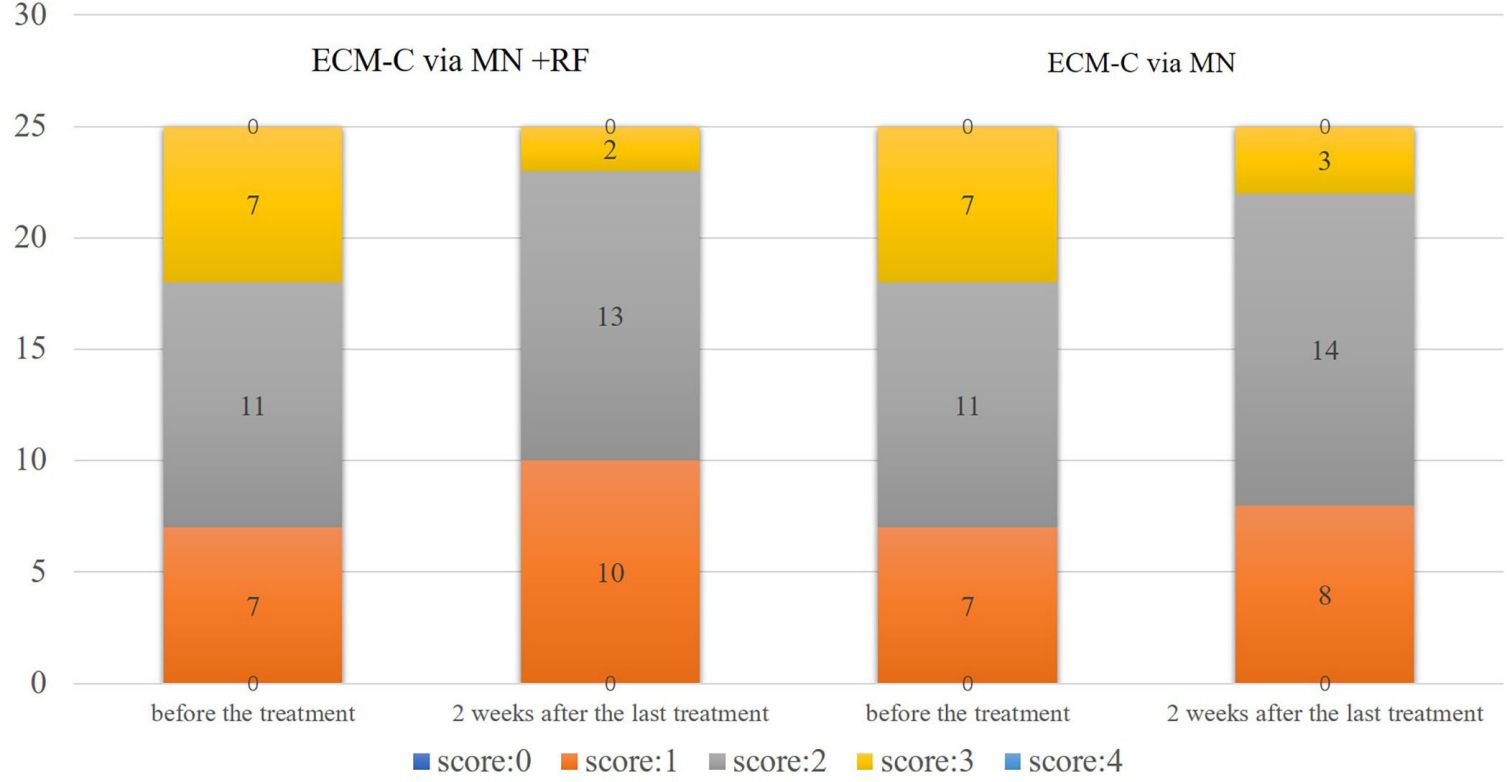
CFGS assessment graph presentation by doctor. CSGF, Crow's Feet Grading Scale; 0 = no wrinkles, 1 = very fine wrinkles, 2 = fine wrinkles, 3 = moderate wrinkles, 4 = severe wrinkles.

Based on CFGS, 7, 11, and 7 participants were assessed for very mild wrinkles, fine wrinkles, and moderate wrinkles, respectively, with both sides at baseline. For the side treated with ECM-C *via* MN, 8, 14, and 3 participants were assessed to have very mild wrinkles, fine wrinkles, and moderate wrinkles, respectively, 2 weeks after the last treatment. For the side treated with ECM-C *via* MN along with RF, 10, 13, and 2 participants were assessed in turn to have very mild wrinkles, fine wrinkles, and moderate wrinkles, and wrinkles scores on both sides decreased significantly after the treatment (*p* = 0.02 and *p* < 0.01). However, there was no statistically significant intergroup difference 2 weeks after the last treatment (*p* = 0.51) in Table 4 and Figure 7.

**Table 4:** CFGS assessment by the doctor.

	**ECM-C via MN**	**ECM-C via MN** + **RF**	***t*** **-value**	***p*** **-value**
**CFGS before the treatment (score)**				
None (0) Very fine wrinkles (1) Fine wrinkles (2) Moderate wrinkles (3) Severe wrinkles (4) Average score	0 7 11 7 0 2 ± 0.76	0 7 11 7 0 2 ± 0.76	0.00	1
**CFGS 2 weeks after the last treatment (score)**				
None (0) Very fine wrinkles (1) Fine wrinkles (2) Moderate wrinkles (3) Severe wrinkles (4) Average score	0 8 14 3 0 1.8 ±0.65	0 10 13 2 0 1.68 ±0.63	0.67	0.51
*t*-value *p*-value	2.45 **0.02**	3.36 **<0.01**		

Statistics are presented as the mean ± SD. CSGF, Crow's Feet Grading Scale. Statistical different *p* values were shown in bold.

### GAIS assessment

**Figure 8:**
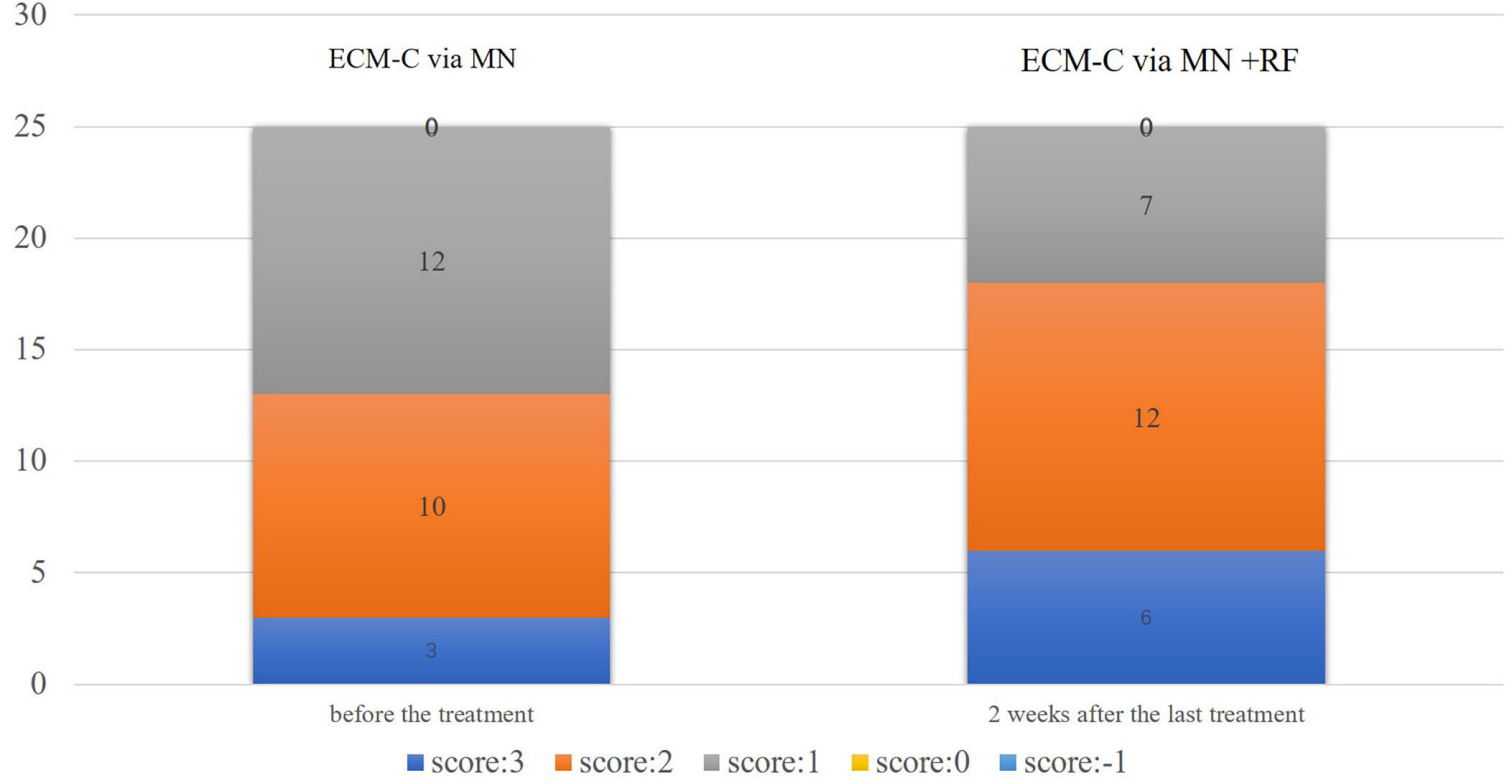
GAIS assessment graph presentation by participants. GAIS, Global Aesthetic Improvement Scale; −1 = worse than the baseline, 0 = no change, 1 = improved, 2 = much improved, 3 = very much improved.

For the side with ECM-C *via* MN, 3 participants assessed “significantly improved,” 10 participants assessed “much improved,” and 12 assessed “improved,” 2 weeks after the last treatment. On the other side, six participants assessed “significantly improved,” 12 participants assessed “much improved,” and seven assessed “improved” according to GAIS. The results are presented in Table 5 and Figure 8.

**Table 5:** GAIS assessment by the study participants.

	**ECM-C via MN**	**ECM-C via MN** + **RF**	***t*** **-value**	***p*** **-value**
**GAIS**	0	0		
Very much improved (3)	3	6		
Much improved (2)	10	12		
Improved (1)	12	7		
No change (0)	0	0		
Worse than baseline (-1)	0	0		
Average score	1.12 ± 0.53	1.40 ± 0.40	−1.93	0.06

Statistics are presented as the mean ± SD. GAIS, Global Aesthetic Improvement Scale.

### Adverse effect assessment

All the participants who were given RF treatment felt warmness and a tolerable amount of heat; some of the participants had transient redness that disappeared after a few minutes to hours. VAS caused by MN treatment ranged from 1–4 score, 4 participants scored 1 (VAS 0), 12 participants scored 2 (VAS 1–3), six participants scored 3 (VAS 4–6), and 3 participants scored 4 (VAS 7–10). There would be a minimal bleeding point after the MN puncturing, which usually stopped in minutes in most cases. Some of them are left with scattered purpura, which is usually self-relieved in 1–3 days. The pain on both sides was not different. The application of ECM-C did not cause any sting or itch.

## Discussion

The appearance of wrinkles, pigmentation, and sagging around the eyes are the first and the most noticeable signs of aging (13). These changes are affected by genetics and environmental factors. The physiological structure and functions of the eye area take the lead in the process of facial aging. Therefore, periorbital aging should be the preferential concern of aging, and cosmetic treatment to rejuvenate the eyes particularly matters. Recently, new theories and techniques against periocular aging without downtime and scar are replacing conventional surgeries that simply remove the excess skin of the eyelid or the bulging fat under the eyes. Until now, the interventions for static wrinkles around the eyes included topical agents, chemical peeling, filler injection, ablative, non-ablative laser, and RF therapy (14–18).

In recent years, comprehensive treatment plans that are more personalized, non-surgical, filler treatment, and micro-invasive treatments have been developed. In this study, to the best of our knowledge, RF was combined with topical ECM-C *via* MN for the first time. We observed the effect on periorbital rejuvenation and investigated the possible synergistic effect of mechanical and heating stimulation on the skin tissue. The principle of combining two or more different non-invasive or minimally invasive modes works as follows: non-invasive treatments such as RF should be given first and the mini-invasive treatment such as MN is given next. The synergy can initiate selective skin injury that leads to tissue remodeling and wound healing process; meanwhile, topical products can be applied to permeate through. This helps to achieve the best patient satisfaction, shortest possible downtime, and relatively low cost (19, 20).

The extracellular matrix is a dynamic 3-dimensional network of macromolecules, including collagens, elastin, and laminins, which provides structural support for the skin (21). It, which is mainly in the dermis, is progressively damaged during aging. This affects the normal organization of the skin and its capacity for repair (22).

The roles of ECM in skin rejuvenation and wound healing are validated, especially its role in repairing the damaged basement membrane in the skin aging process has attracted more attention (23). The basement membrane is a highly specialized ECM that tightly connects the epidermis and dermis. During the skin aging process, the structure and function of the skin basement membrane undergo many changes. For example, type IV collagen degradation and dense layer destruction contribute to the damage of the basement membrane function, and subsequent melanin deposition in the dermis is considered a key feature of refractory melasma (24). At present, a wide range of products is available in the cosmetic market worldwide with diverse functions focusing on anti-wrinkle and whitening. Repairing the elastic structure of aging cells *via* internal means, adjusting the composition of the ECM, and restoring the elastic structure of the skin are inevitable trends for a new generation of anti-aging products. ECM-C applied in this study is a biomimetic basement membrane consisting of various components such as type IV and VII collagens, adhesion proteins, and hyaluronic acid, all of which are pure, natural, and biologically active and directly replenish the basement membrane. The nutrients in the dermis can reach the epidermis smoothly, promote the proliferation and differentiation of basal keratinocytes, and provide “soil” and “signal” for tissue repair and regeneration.

Microneedle therapy, also known as collagen induction therapy (CIT) or transdermal CIT, is an increasingly popular procedure for skin regeneration. It promotes skin regeneration by creating small puncture wounds in the epidermis and dermis. Such injuries trigger the wound healing cascade and release several growth factors to promote regeneration (25). In addition, MN therapy creates dense micron-sized pathways, causing a significant widening of the follicular infundibulum by 47% (26). This may help increase skin permeability and thus nutrient absorption. Recently, clinicians have used platelet-rich plasma for enhancing cosmetic effects (27). The MN has also shown promising effects in the treatment of androgenic alopecia, increasing hair regrowth in patients who previously did not respond well-to conventional treatments with minoxidil and finasteride (28, 29).

Based on the above theory and clinical practice, we combined the MN with the ECM-C for periorbital rejuvenation. The MN facilitated ECM-C absorption in the punctured area, whereas the ECM-C renewed the skin microenvironment and accelerated the regenerative effects of the MN while repairing the basement membrane. Our findings showed that significant improvements in skin hydration, wrinkles, skin elasticity, and thickness were observed after the treatment. However, no significant difference was observed before and after the treatment regarding melanin, erythema, and TEWL. This also indicates that the MN combined with the ECM-C did not increase the risks of pigmentation and destruction of the skin barrier. Wamsley et al. applied the MN for facial photo-aging treatment four times a month. In the 2nd month after the end of the treatment, the levels of inflammatory factors, such as interleukin (IL)-1β, tumor necrosis factor α, and IL-6, were higher than the baseline levels. However, these changes in inflammation were not statistically significant, and the blood flow did not increase after MN therapy (30). The MN may increase tissue oxygen levels, thereby stimulating fibroblasts to differentiate into myofibroblasts, thus inhibiting neovascularization (24). In this study, the patients could return to normal within 3–5 days after MN therapy, which is consistent with previous studies (31). No significant change was observed in the erythema value detected 2 weeks after the end of the treatment, indicating that MN therapy neither increased the inflammatory response nor caused the persistence of erythema.

Monopolar RF is the first RF system introduced for aesthetic purposes, which was approved by the U.S. Food and Drug Administration in 2002 for the treatment of facial skin wrinkles (32). Since then, various RF instruments have been widely used in cosmetic dermatology, and RF has become a viable option for skin regeneration and tightening. Compared with laser photothermal energy, RF does not rely on chromophores; hence, it is considered suitable for all skin types (9). Maintaining the skin surface temperature below 42–45°C is essential for the safety of RF treatment because the threshold for epidermal burns is 44°C (33). RF overcomes the following limitations in wrinkle removal with laser or intense pulsed light: the insufficient penetration depth and post-treatment pigmentation because of melanocyte irritation in the basal layer of the epidermis. Moreover, it also guarantees the safe heating of the epidermal skin by targeting dermal collagen (34). In this study, a sliding treatment head of monopolar RF was used to treat the patient's eye area on one side, and a non-contact infrared thermometer was used to monitor the temperature of the treatment area, keeping the surface temperature around 40°C to achieve the best treatment effect. After the treatment with monopolar RF, most participants experienced mild erythema, which disappeared within 1–3 h after the procedure. RF emits high-frequency electromagnetic waves to act on collagen that is deeply embedded in the dermis layer. As a result, the tissue arrangement becomes more compact, and the skin is tightened with wrinkles reduced. MN therapy can also promote elastic fiber formation, collagen deposition, and dermal thickening (35). In this study, the applications of the two were combined, that is, the MN and ECM-C were introduced following the RF treatment. The results showed that the combined treatment group showed better effects than the ECM-C *via* MN group regarding wrinkle improvement, skin elasticity, and skin thickness. On the one hand, we speculated that the combined applications of the two modalities on the same day could promote the proliferation of more collagen fibers than single therapy; after the mechanical stimulation by the MN and thermal stimulation by RF, more fibroblasts were activated to regenerate more collagen. On the other hand, numerous pricks of the MN and ECM-C penetrated through this minimal tunnel to the dermis layer to fill the dermis with more supporting and water-retaining components. In RF treatment, a critical temperature of 65–75°C was generated in the deep layer of the dermis and the fiber interval in its deep part, and a temperature of 35–45°C was generated in the epidermis (17). However, studies have shown that the combined application of RF and fillings on the same day would not degrade the fillings, affect the durability of the fillings, or lead to increased adverse reactions (36). In addition, RF microporation assists macromolecular substances to penetrate the skin barrier. The stratum corneum of the epidermis is a protective barrier of the skin, which prevents the passage of proteins, peptides, and water-soluble macromolecular substances. RF can change the barrier function of the stratum corneum, thereby facilitating the passage of macromolecular substances (37). Therefore, the approach of the ECM-C *via* MN after RF did not affect the efficacy of its active ingredients and promoted the transepidermal absorption of the active ingredients. The reason behind this is the rapid restoration of wrinkle appearance presented within the short period of 10 weeks (from baseline to 2 weeks after the last treatment).

To summarize, all the participants were satisfied with the improvement in periorbital wrinkles. Each treatment was safe, effective, and well-tolerated. Combined treatments were more effective in improving periorbital wrinkles. RF combined with the ECM-C *via* the MN takes advantage of RF tightening, MN resurfacing, and transepidermal drug delivery. It overcomes the shortcomings and deficiencies of conventional optical monotherapy with a promising prospect of rejuvenating the area around the eyes. A study reported that new collagen fibers are most obvious 6 months after RF treatment (37). Interestingly, the objective assessment showed that periocular wrinkles, skin elasticity, and skin thickness improved on the side treated with the ECM-C *via* the MN and RF than on the side treated with the ECM-C *via* the MN alone. However, the subjective CFGS and GASI scores assessed by two different doctors showed no statistically significant difference between the two sides. The two potential reasons for this disagreement are given as follows: one is the relatively short period of the follow-up in this study, and the other reason is what we see with our eyes was more roughly compared with this objective test, which helps in observing more details. Therefore, we might neglect some slight alterations for a bigger view with the naked eyes. There are also some limitations of this study; first, the number of recruited volunteers was small. Second, the follow-up time is relatively short, which makes it uncertain about the long-term effect of anti-periorbital wrinkles. Hence, a large sample size and longer follow-up time would be necessary to verify this rejuvenation outcome.

## Data availability statement

The data generated for this study are available upon reasonable request to the corresponding author.

## Ethics statement

The studies involving human participants were reviewed and approved by institutional review board of Beijing Friendship Hospital Ethics Committee. The patients/participants provided their written informed consent to participate in this study. Written informed consent was obtained from the individual(s) for the publication of any potentially identifiable images or data included in this article.

## Author contributions

FZ was in charge of the study design, assessment of CFGS, and editing of the manuscript. HC contributed to the treatment, subjective tests, assessment of GAIS, and writing of this manuscript. RZ helped with the assessment of CFGS, data analyses, and manuscript writing. All authors contributed to the article and approved the submitted version.

## Conflict of interest

The authors declare that the research was conducted in the absence of any commercial or financial relationships that could be construed as a potential conflict of interest.

## Publisher's note

All claims expressed in this article are solely those of the authors and do not necessarily represent those of their affiliated organizations, or those of the publisher, the editors and the reviewers. Any product that may be evaluated in this article, or claim that may be made by its manufacturer, is not guaranteed or endorsed by the publisher.
